# Cardiac Catheterization in a Patient with Obstructive Hypertrophic Cardiomyopathy and Syncope

**DOI:** 10.5935/abc.20170098

**Published:** 2017-09

**Authors:** Rafael Amorim Belo Nunes, Henrique Barbosa Ribeiro, Luiz Junya Kajita, Fábio Antônio Gaiotto

**Affiliations:** Instituto do Coração - Hospital das Clínicas - Faculdade de Medicina - Universidade de São Paulo (USP), São Paulo, SP - Brazil

**Keywords:** Cardiomyopathy, Hypertrrophic, Cardiac Catheterization, Syncope

A 35-year-old man sought medical care for recurrent syncope episodes related to moderate exertion in the past 2 months. Upon physical examination, the presence of a rude systolic murmur on the left sternal border was identified. The echocardiogram disclosed a moderate increase in the left atrium and significant hypertrophy of the interventricular septum with an estimated maximum diastolic diameter of 31 mmHg and a maximum left ventricular outflow tract gradient of 56 mmHg. The 24-hour Holter assessment showed the presence of frequent ventricular extrasystoles and an episode of nonsustained ventricular tachycardia. He was prescribed metoprolol 50 mg daily and, based on the high risk of sudden death, received an implantable cardioverter-defibrillator.

Despite the progressive increase in beta-blocker doses, the patient remained quite symptomatic with daily episodes of lipothymia and angina pectoris at minor exertion. Invasive strategy to reduce the intraventricular gradient was planned and the patient underwent a hemodynamic study to better assess the coronary and interventricular septum anatomy. The coronary angiography showed extrinsic compression of the first diagonal branch and septal arteries (Panel A). Simultaneous ventriculography of both ventricles disclosed significant hypertrophy of the medial and basal portions of the interventricular septum (Panel B) with left ventricular outflow tract obstruction (Panel C). The isoproterenol infusion during manometry resulted in increased intraventricular gradient from 30 mmHg to 130 mmHg, which revealed an important dynamic obstructive component. The patient was submitted to septal myectomy with no complications, with a significant reduction in the intraventricular gradient.

## Figures and Tables

**Figure 1:**
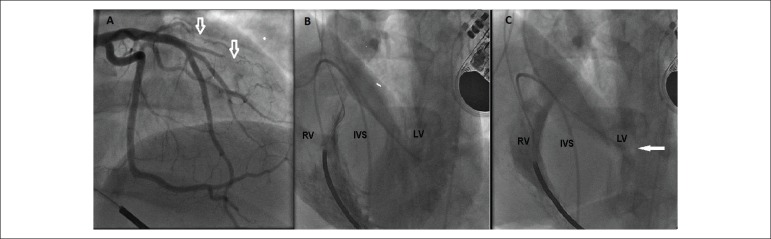
Panel A, extrinsic compression of the diagonal branch and septal branches (white arrows). Panel B, ventriculography of the right ventricle (RV) and left ventricle (LV), showing significant interventricular septal (IVS) hypertrophy during diastole and Panel C, LV outflow tract obstruction during end-systole.

